# Tracheobronchial Foreign Body Aspiration Diagnosed with Electrical Impedance Tomography

**DOI:** 10.1155/2021/9951838

**Published:** 2021-11-02

**Authors:** Robert D. Guglielmo, Robinder G. Khemani

**Affiliations:** ^1^Division of Pediatric Critical Care, Department of Pediatrics, Loma Linda University Children's Hospital, Loma Linda University School of Medicine, Loma Linda, CA, USA; ^2^Department of Anesthesiology Critical Care Medicine, Children's Hospital Los Angeles (CHLA), Keck School of Medicine of the University of Southern California (USC), Los Angeles, CA, USA

## Abstract

**Background:**

Foreign body aspiration (FBA) in children has a high morbidity, and early diagnosis is the key for preventing acute and chronic respiratory complications. To diagnose FBA, commonly used imaging modalities have limited negative predictive value, and rigid bronchoscopy remains as the gold standard. We present a case where the diagnosis of FBA was made in a novel way with electrical impedance tomography (EIT). *Case Presentation*. A 19-month-old previously healthy boy was admitted with a clinical diagnosis of respiratory failure secondary to bronchiolitis. Chest X-ray showed bilateral lung hyperinflation. He enrolled in a research study which used EIT to measure the effects of high flow nasal cannula (HFNC) on minute ventilation in children with bronchiolitis. On initiation, the patient had near-normal right lung ventilation (98%) and near-absent left lung ventilation (2%). We discontinued the study and alerted the medical team that we suspected FBA. Further imaging (lateral decubitus films and lung ultrasounds) was also obtained, but was not diagnostic. Rigid bronchoscopy was performed and showed a peanut occluding the left mainstem bronchus (LMB). The peanut was removed followed by complete resolution of the patient's symptoms.

**Conclusions:**

We believe this is the first reported case of FBA diagnosed via EIT. EIT has been shown to be a useful but underutilized technology for diagnosing respiratory disease. While FBA remains a relatively common cause of morbidity and mortality in children less than age four, early diagnosis remains difficult and requires vigilance. This case illustrates the challenges of relying on chest films and ultrasound to assist with diagnosis and suggests that EIT in combination with a thorough history and physical exam can be used to confirm the presence of FBA.

## 1. Introduction

Foreign body aspiration (FBA) is a high morbidity diagnosis for children. FBAs are commonly seen in the emergency room (ER) and in pediatric intensive care unit (PICU). Early diagnosis of FBA is critical to prevent acute and chronic respiratory complications including but not limited to respiratory failure, asphyxia, pneumonia, atelectasis, bronchospasm, air trapping, pneumothorax, bacterial abscesses, and cardiopulmonary arrest [1]. FBA is a difficult diagnosis to make and is commonly missed by experienced physicians, with some centers quoting as high as 44% in delayed diagnosis [2]. Tests used to aid in FBA diagnosis include X-rays and ultrasound, but they are limited by their negative predictive values, so children with suspected FBAs often require general anesthesia and a rigid bronchoscopy. We present a case where the diagnosis of FBA was made in a novel way with electrical impedance tomography (EIT), a noninvasive invasive imaging modality that requires no radiation and can be available quickly at the bedside. EIT uses surface electrodes encased in flexible bands to form real-time tomographic images of a body area. The bands go circumferentially around a body area (in this case, the patient's thorax), and the attached EIT machine uses an electrical current to generate images via impedance differences.

## 2. Case Presentation

**Figure 1:**
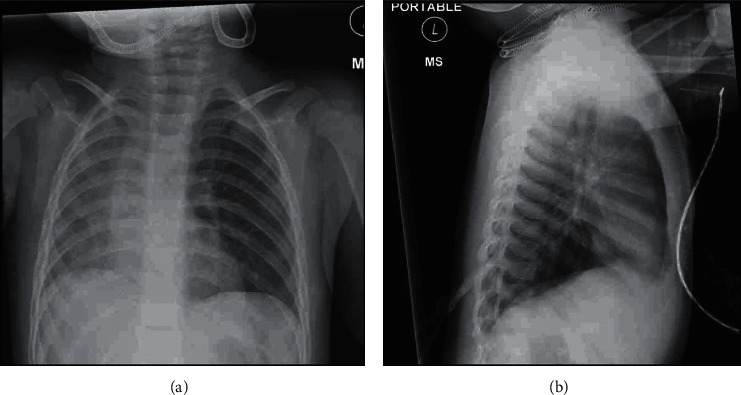
Erect (a) anteroposterior and (b) lateral chest X-rays taken for this patient prior to using the EIT device. These films were read by the attending pediatric radiologist: “On the lateral view both the lungs appear hyperinflated. There is relative decreased lung volume on the frontal view which is felt to be related to patient rotation.”

**Figure 2:**
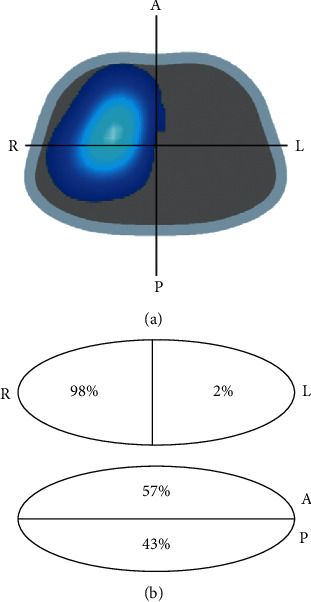
(a) EIT ventilation map for the patient showing right lung ventilation and (b) the EIT ventilation distribution showing 98% of ventilation occurring on the right lung. Figure axes include anterior lung fields (A), posterior lung fields (P), right lung (R), and left lung (L).

A 19-month-old previously healthy boy presented in acute respiratory failure with a diagnosis of bronchiolitis and reactive airways. This child had rhinovirus/enterovirus diagnosed via respiratory virus panel (RVP). The RVP, along with his clinical exam, led the medical teams in both the ER and PICU to a clinical diagnosis of bronchiolitis and presumed asthma. The patient clinically deteriorated on bronchodilators, steroids, and high flow nasal cannula (HFNC). On exam, he had asymmetric breath sounds and respiratory distress. Erect anteroposterior and lateral chest X-rays (Figure 1) were read by the pediatric radiologist as “…both lungs appear hyperinflated. …decreased lung volume on the frontal view which is felt to be related to patient rotation.” The patient was enrolled in a prospective research study which used EIT to measure the effects of HFNC on minute ventilation in children with bronchiolitis. On initiation of EIT (Figure 2, Video 1), he had near-normal right lung ventilation (98%) and nearly absent left lung ventilation (2%). We obtained more history and learned the patient had worsened respiratory distress after coughing while eating days prior. We discontinued the study and alerted the medical team that we suspected foreign body aspiration (FBA) and that rhinovirus/enterovirus was a parallel diagnosis.

**Figure 3:**
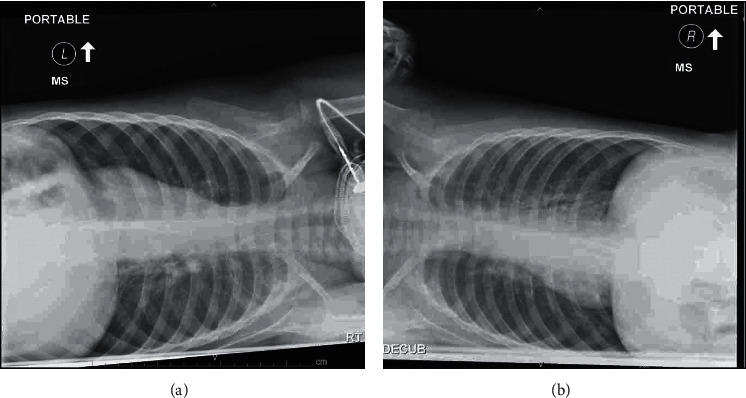
(a) Right and (b) left lateral decubitus chest X-rays taken after the EIT device was used in attempt to confirm diagnosis of FBA by more traditional means. The attending pediatric radiologist read these images: “Both lungs are hyperinflated on the decubitus view without significant asymmetry which suggests air trapping secondary to bronchiolitis.”

The medical team called the attending pediatric radiologist who reexamined the prior anteroposterior and lateral X-rays. The radiologist confirmed that the images were consistent with hyperinflation due to bronchiolitis, but we agreed to order right and left lateral decubitus chest X-rays and chest ultrasounds to further investigate for FBA. The decubitus films (Figure 3) were read: “Both lungs are hyperinflated on the decubitus view without significant asymmetry which suggests air trapping secondary to bronchiolitis.” Lung ultrasounds were read: “Motion was seen at the bilateral hemidiaphragms, with more motion of the right hemidiaphragm as compared to left.”

**Figure 4:**
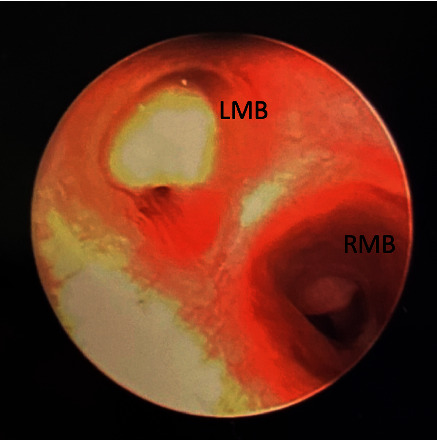
Images taken from diagnostic bronchoscopy, displaying a peanut causing near-complete occlusion of the left mainstem bronchus (LMB) and granulation tissue in the right mainstem bronchus (RMB). The RMB granulation tissue suggesting the peanut had recently moved, potentially after coughing, leading to the acute deterioration. The peanut was subsequently removed and the patient improved rapidly.

Clinical suspicion for FBA remained high due to the EIT test results and the patient's asymmetric physical exam. Otolaryngology was consulted and subsequently performed a rigid bronchoscopy, where a peanut was found to be occluding the left mainstem bronchus (LMB) (Figure 4). Granulation tissue was seen in the right mainstem bronchus (RMB), which suggests that the peanut was originally in the RMB, but had recently moved to the LMB, potentially after coughing, leading to the patient's acute deterioration. The peanut was extracted followed by complete resolution of the patient's symptoms. The patient was discharged home the subsequent day.

## 3. Discussion

We believe this is the first reported case of FBA diagnosed via EIT. While FBA remains a relatively common cause of morbidity and mortality in children less than age four, early diagnosis remains difficult and requires vigilance [1, 3]. While diagnostics such as anteroposterior X-rays, decubitus films, and lung ultrasound can sometimes be useful, they have limited negative predictive value, and rigid bronchoscopy remains the gold standard to diagnose FBAs [4]. Rigid bronchoscopy is an invasive procedure requiring general anesthesia and has significant risk [5], while EIT is an underutilized technology that requires no radiation, no sedation, and can be applied easily at the patient bedside. EIT has not before been used for FBA diagnosis, but has been used for determining proper one lung ventilation after endotracheal intubation [6, 7].

The EIT device used in this study is FDA approved for use in adults and was approved by the CHLA Institutional Review Board (CHLA-19-00341) for the intended research study. While in this case, the Enlight® 1800 device was used, and there are now several companies which provide EIT technology at the bedside. EIT is currently underutilized clinically, but the set-up process takes less than 5 minutes and requires soft-flexible bands to be placed circumferentially around the patient's chest and the EIT device. This is the technology which could easily be performed in an outpatient or ED setting if they have an EIT device.

This case itself illustrates the challenges of relying on current more commonly used imaging modalities such as radiographic films and ultrasounds to assist with the diagnosis of FBA. This patient in particular had a parallel diagnosis of rhinovirus/enterovirus which made spotting the clinical signs of an FBA more challenging. This case suggests that EIT in combination with a thorough history and physical exam can be used to confirm the presence of FBA.

## Data Availability

All data generated or analyzed during this study are included within the article.

## Supplementary Materials

Supplementary Materials Video 1: EIT video taken shortly after connecting the EIT device, displaying right lung ventilation. The right lung is on the left side of the image, and the left lung is on the right side of the image (layout matches that of a traditional transverse cross-section of a computed tomography scan). Google Drive link: https://drive.google.com/file/d/1jMtLwqx2NC9oDg2ke4eRFDGAjbqWXyki/view?usp=sharing.

## References

[1] Saquib Mallick M., Rauf Khan A., Al-Bassam A. (2005). Late presentation of tracheobronchial foreign body aspiration in children. *Journal of Tropical Pediatrics*.

[2] Huang Z., Liu D., Zhong J., Zhou J. (2013). Delayed diagnosis and treatment of foreign body aspiration in China: the roles played by physician inexperience and lack of bronchoscopy facilities at local treatment centers. *International Journal of Pediatric Otorhinolaryngology*.

[3] Haddadi S., Marzban S., Nemati S. (2015). Tracheobronchial foreign-bodies in children; A 7 Year retrospective study. *Iranian Journal of Otorhinolaryngology*.

[4] Bhalodiya N., Supriya M., Patel S. (2006). Foreign body inhalation in children: decisive factors for carrying out bronchoscopy. *Indian Journal of Otolaryngology and Head & Neck Surgery*.

[5] Maddali M. M., Mathew M., Chandwani J., Alsajwani M. J., Ganguly S. S. (2011). Outcomes after rigid bronchoscopy in children with suspected or confirmed foreign body aspiration: a retrospective study. *Journal of Cardiothoracic and Vascular Anesthesia*.

[6] Schmölzer G. M., Bhatia R., Davis P. G., Tingay D. G. (2013). A comparison of different bedside techniques to determine endotracheal tube position in a neonatal piglet model. *Pediatric Pulmonology*.

[7] Steinmann D., Stahl C. A., Minner J. (2008). Electrical impedance tomography to confirm correct placement of double-lumen tube: a feasibility study. *British Journal of Anaesthesia*.

